# Continuous Passive Motion Promotes and Maintains Chondrogenesis in Autologous Endothelial Progenitor Cell-Loaded Porous PLGA Scaffolds during Osteochondral Defect Repair in a Rabbit Model

**DOI:** 10.3390/ijms20020259

**Published:** 2019-01-10

**Authors:** Hsueh-Chun Wang, Tzu-Hsiang Lin, Nai-Jen Chang, Horng-Chaung Hsu, Ming-Long Yeh

**Affiliations:** 1 Department of Biomedical Engineering, National Cheng Kung University, Tainan City 70101, Taiwan; whc32002@hotmail.com (H.-C.W.); jeff.qbmc@yahoo.com.tw (T.-H.L.); 2 Department of Sports Medicine, Kaohsiung Medical University, Kaohsiung City 80708, Taiwan; njchang@kmu.edu.tw; 3 Department of Orthopedics, China Medical University Hospital, Taichung 40447, Taiwan; d4749@mail.cmuh.org.tw; 4 Medical Device Innovation Center, National Cheng Kung University, Tainan City 70101, Taiwan

**Keywords:** continuous passive motion, osteochondral tissue engineering, PLGA scaffolds, epithelial progenitor cells

## Abstract

Continuous passive motion (CPM) is widely used after total knee replacement. In this study, we investigated the effect of CPM combined with cell-based construct-transplantation in osteochondral tissue engineering. We created osteochondral defects (3 mm in diameter and 3 mm in depth) in the medial femoral condyle of 36 knees and randomized them into three groups: ED (empty defect), EPC/PLGA (endothelial progenitor cells (EPCs) seeded in the poly lactic-co-glycolic acid (PLGA) scaffold), or EPC/PLGA/CPM (EPC/PLGA scaffold complemented with CPM starting one day after transplantation). We investigated the effects of CPM and the EPC/PLGA constructs on tissue restoration in weight-bearing sites by histological observation and micro-computed tomography (micro-CT) evaluation 4 and 12 weeks after implantation. After CPM, the EPC/PLGA construct exhibited early osteochondral regeneration and prevention of subchondral bone overgrowth and cartilage degeneration. CPM did not alter the microenvironment created by the construct; it up-regulated the expression of the extracellular matrix components (glycosaminoglycan and collagen), down-regulated bone formation, and induced the biosynthesis of lubricin, which appeared in the EPC/PLGA/CPM group after 12 weeks. CPM can provide promoting signals during osteochondral tissue engineering and achieve a synergistic effect when combined with EPC/PLGA transplantation, so it should be considered a non-invasive treatment to be adopted in clinical practices.

## 1. Introduction

Osteochondral tissue is heterogeneous, consisting of smooth cartilage that lines the articulating surface and underlying subchondral bone. This complexity impedes the tissue restoration after an injury to the joint or a disease. Current clinical treatments for osteochondral defects include microfracture surgery, autologous chondrocyte implantation (ACI), and osteochondral transplantation (OCT). However, they present problems such as an inability to produce hyaline cartilage, poor integration with the surrounding cartilage, and gradual deterioration of the repaired tissue.

ACI was first used in 1994 [1] to treat cartilage defects. It is a durable and beneficial treatment for chondral knee defects as patients have reported better pain relief and greater functional improvement than with other procedures [1,2,3,4]. However, there are still obstacles to overcome, such as a long time for cell expansion in vitro [5], chondrocyte dedifferentiation [6,7], and upward migration of the subchondral bone [8,9]. ACI cannot repair the osteochondral interface or the cartilage to full-thickness. To overcome this, a new generation ACI—named matrix-induced ACI (MACI)—is required [10]. MACI allows cultured chondrocytes to penetrate the 3-dimensional scaffold and repair critical lesions in the cartilage. However, in MACI, a two-step surgery is necessary first to obtain the chondrocytes and then to complete the implantation procedure.

Mesenchymal stem cells (MSCs), such as adipose tissue-derived MSCs [11] and bone marrow-derived MSCs, are promising cell-types that could be used in MSC-based cartilage tissue engineering. However, MSCs are prone to undergoing endochondral ossification [12]. Previously, we demonstrated that autologous endothelial progenitor cells (EPCs) seeded into poly lactic-co-glycolic acid (PLGA) scaffolds created a healthy microenvironment for osteochondral regeneration [13] since EPCs secreted paracrine factors that induced hyaline cartilage and underlying bone regeneration in osteochondral defects.

EPCs were discovered by Asahara et al. in 1997 [14] and were defined as a special type of stem cells. They have therapeutic potential for regeneration and vasculogenesis of different tissues either directly through cell fusion or indirectly through paracrine mechanisms. EPC-based therapies are used in cardiovascular diseases [15], limb ischemia [16,17], vascular repair [14,18], bone regeneration [19,20,21], and acute lung injury [22,23]. Furthermore, EPCs can be extracted from the peripheral blood which avoids invasive procedures and severe postoperative pain. A delivery system that incorporates EPCs into PLGA scaffolds could facilitate the nutrition from the subchondral bone to the cartilage and help to regenerate the hyaline cartilage [13]. However, homeostasis maintenance is still a concern.

Continuous passive motion (CPM) is an early postoperative intervention for patients undergoing total knee arthroplasty (TKA) that increases trans-synovial transport to intra-articular tissues by producing sinusoidal pressure [24]. This procedure clears the joint from low-molecular-weight solutes and improves the health of the meniscus and articular cartilage. In a recent study, we demonstrated that a combination of early CPM and acellular PLGA implantation induces significant hyaline cartilage regeneration and trabecular boney deposition when compared to immobilization and intermittent active motion treatments during osteochondral regeneration in a rabbit knee joint model [25]. Importantly, CPM can improve cartilage and joint health by stimulating the synthesis of lubricin, which is encoded by the *proteoglycan 4* (*PRG4*) gene in chondrocytes [26].

Lubricin is a chondroprotective glycoprotein that is secreted by the chondrocytes of the superficial zone of the articular cartilage [27] and functions as a boundary lubricating molecule on the surface of articular cartilage, reducing friction and wear [28,29,30]. Lubricin acts as a marker of chondrocyte senescence and/or osteoarthritis (OA) [31]. It can be upregulated by stimuli that mimic articular motion to promote regeneration of the functional articular surface-synovial interface [32]. Furthermore, Musumeci et al. demonstrated greater lubricin production in elderly rats that endured adequate physical exercise and mechanical stimulation than in unexercised adult rats [33].

Since lubricin plays such an important role in maintaining the homeostasis of the articular cartilage and CPM could stimulate its synthesis, we hypothesized that early CPM would enhance the in vivo response to the autologous EPC/PLGA construct, increase its ability to regenerate the osteochondral tissue, and create a conservatory superficial zone in the cartilage of a weight-bearing defect in a rabbit knee. The combined effects could lead to the repair of tissue-specific osteochondral defects within the cartilage and subchondral bone—without a supplement of exogenous growth factors in the early stages—and further prevent cartilage degeneration.

## 2. Results

### 2.1. Characteristics of the 3D PLGA Scaffolds

**Figure 1:**
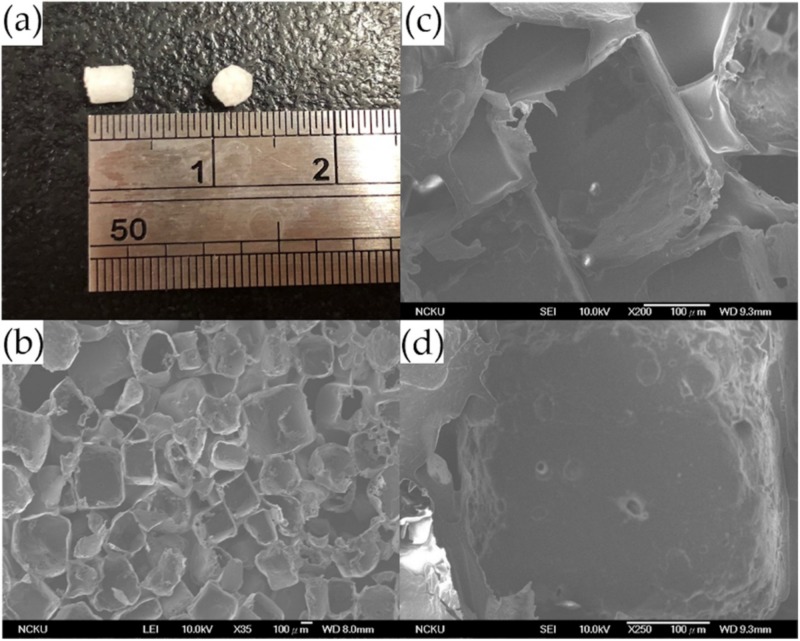
Characteristics of the PLGA scaffold. PLGA sponge scaffold (**a**), and scanning electron microscopy images of the PLGA structure under different magnifications: x35 (**b**); x 200 (**c**); and x 250, the interconnectivity of the PLGA scaffold (**d**). Scale bar 100 μm.

The porous PLGA scaffolds fabricated in this study were 3 mm in diameter and 3 mm in height as shown in (Figure 1a), the inner porous structure, interconnectivity, and honeycomb-like structures were observed by scanning electron microscopy (Figure 1b–d). The diameter of the pores ranged from 300 to 500 μm (Figure 1c,d), and the porosity was over 90% based on a calculation of the difference between the bulk density and the true density.

### 2.2. Tracing the Location of EPCs by an In Vivo Imaging System (IVIS) and Spectrum CT Analyses In Vivo

**Figure 2:**
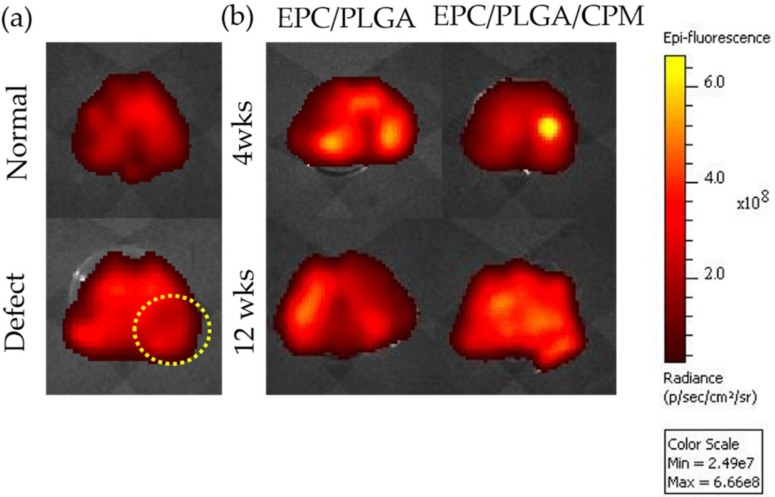
Location of endothelial progenitor cells (EPCs) after transplantation for the normal and defect only groups; the yellow dotted circle indicates the defect site (**a**). Localization of EPCs by CM-Dil 4 and 12 weeks after PLGA scaffold transplantation with or without continuous passive motion (CPM) intervention (**b**). The bioluminescence was determined by in vivo imaging system (IVIS) measurements in the dissected tissue of the femurs of the EPC/PLGA and EPC/PLGA/CPM groups.

The bioluminescence values of the EPC/PLGA and EPC/PLGA/CPM groups after 4 weeks were similar, with both around 6.3 photons/sec/cm^2^/steradian (p/s/cm^2^/sr). The bioluminescence was conspicuous in the lesioned core 4 weeks after implantation in the EPC/PLGA/PLGA group (Figure 2). In contrast, there were three spots of diffuse bioluminescence that seemed to flow around the joint capsule in the EPC/PLGA group. After 12 weeks, the bioluminescence of the two groups decreased, but was still detectable on the surface of the cartilage (Figure 2), the value was about 6 p/s/cm^2^/sr.

### 2.3. Macroscopic Observations and Quantitative Scores

#### 2.3.1. Gross Appearance

**Figure 3:**
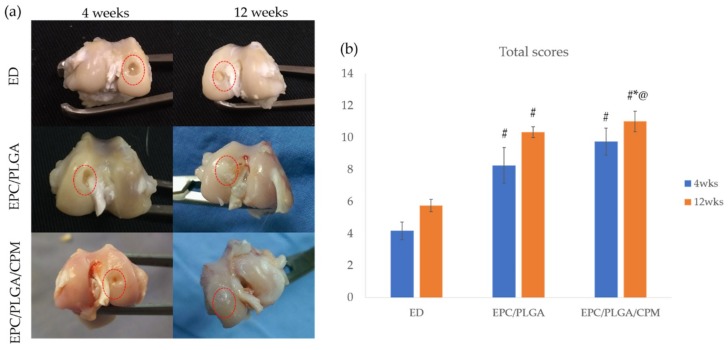
Macroscopic tissue regeneration in different groups. Gross appearance of the articular cartilage defects at 4 and 12 weeks post-operation, the red dotted circles indicate the defect sites (**a**). Qualitative scores of the gross appearances of the empty defect (ED), EPC/PLGA, and EPC/PLGA/CPM groups at 4 and 12 weeks post-operation (**b**). *: compared to 4 weeks post-operation, *p* < 0.05; #: compared to the ED at the same time point, *p* < 0.01; @: compared to the EPC/PLGA group at the same time point, *p* < 0.05.

We did not see inflammatory reactions or joint contractures during the whole postoperative period in any group. After 4 weeks, the EPC/PLGA and EPC/PLGA/CPM groups had reparative tissue at the margins of the defect, the central areas of the defects were depressed. CPM improved the amount of repaired tissue. The injured regions were obviously concave in the empty defect (ED) group (Figure 3a).

At 12 weeks, the EPC/PLGA/CPM group exhibited neo-cartilage-like tissue and a transparent, smooth, and shiny articular surface resembling the surroundings of normal hyaline-like cartilage. The EPC/PLGA group also showed a smooth surface in the articular cartilage, but some mild, whitish fibrous tissue was present (Figure 3a).

#### 2.3.2. Quantitative Scores

At 4 weeks, the total scores in the EPC/PLGA/CPM (9.75 ± 0.84) and EPC/PLGA (8.25 ± 1.12) groups were not significantly different (*p* < 0.20), but they were significantly higher than those of the ED group (4.17 ± 0.54) (*p* < 0.01, for both) (Figure 3b).

At 12 weeks, the score of the EPC/PLGA/CPM group (11 ± 0.63) was significantly higher than those of the ED (5.75 ± 0.39) and EPC/PLGA (10.33 ± 0.33) groups (*p* < 0.01, *p* = 0.008, respectively) (Figure 3b). The score of the EPC/PLGA group was also significantly higher than that of the ED group (*p* < 0.01) (Figure 3b).

### 2.4. Micro-CT Analysis

#### 2.4.1. Findings after 4 Weeks

**Figure 4:**
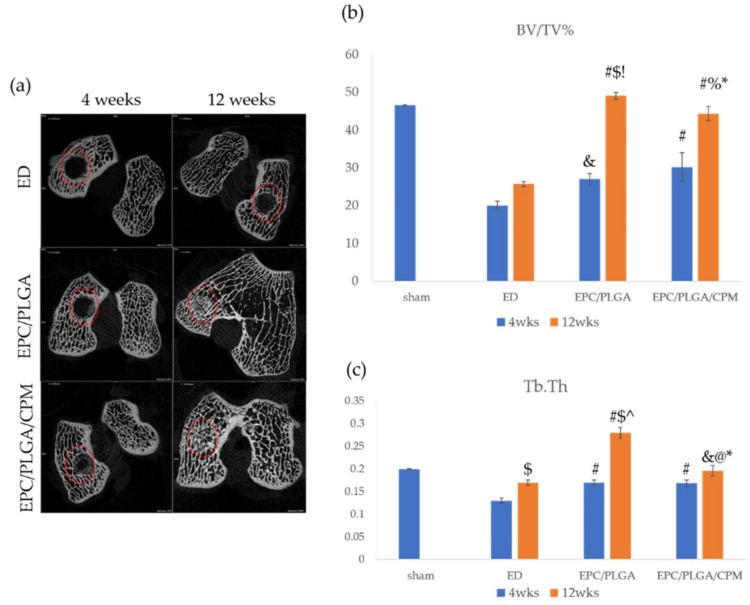
Bone regeneration over time. Bone assessment of 2D micro-CT images, the red dotted circles indicate the defect sites. (**a**); ratio of bone volume to tissue volume (BV/TV) (**b**); and thickness of trabecular bone (Tb.Th) (**c**). *: compared to 4 weeks post-operation (*p* < 0.05); $: compared to 4 weeks post-operation, (*p* < 0.01); &: compared to ED at the same time point (*p* < 0.05); #: compared to ED at the same time point (*p* < 0.01); %: compared to EPC/PLGA at the same time point (*p* < 0.05); @: compared to EPC/PLGA at the same time point (*p* < 0.01); !: compared to sham at 12 weeks, (*p* < 0.05); ^: compared to sham at 12 weeks (*p* < 0.01).

During the 4th week after implantation, a newly formed osseous matrix developed from the edge of the defect toward the central area in the EPC/PLGA and EPC/PLGA/CPM groups (Figure 4a). The bone volume per tissue volume (BV/TV) and thickness of trabecular bone (Tb.Th) values of the EPC/PLGA/CPM (31.04 ± 3.83, *p* < 0.01; 0.17 ± 0.007, *p* = 0.007, respectively) and EPC/PLGA (27 ± 1.52, *p* = 0.01; 0.17 ± 0.005, *p* = 0.004, respectively) groups were significantly different to those of the ED group (20 ± 1.15; 0.13 ± 0.005, respectively) (Figure 4b,c). There was no significant difference between the BV/TV and Tb.Th values of the EPC/PLGA/CPM and the EPC/PLGA groups (Figure 4b,c). Every group mentioned above showed significantly lower BV/TV and Tb.Th values (*p* < 0.05) than the sham group (Figure 4b,c).

#### 2.4.2. Findings at 12 Weeks

During the 12th week after implantation, the EPC/PLGA group exhibited the highest BV/TV and Tb.Th values (49.02 ± 0.94, 0.28 ± 0.012, respectively); both values were significantly higher than those of the ED (25.67 ± 0.67, *p* < 0.01; 0.17 ± 0.006, *p* < 0.01, respectively) and EPC/PLGA/CPM (44.33 ± 1.20, *p* = 0.02; 0.196 ± 0.011, *p* < 0.01, respectively) groups. However, the values of the EPC/PLGA/CPM group were not significantly different to those of the sham group (BV/TV: 46.55 ± 0.15, *p* = 0.07; Tb.Th: 0.2 ± 0.001, *p* = 0.38) while the values of the ED group were significantly lower than those of the sham group (BV/TV, *p* = 0.03; Tb.Th, *p* < 0.01) (Figure 4b,c).

#### 2.4.3. Comparison between the Micro-CT Analysis at 4 and 12 Weeks

We compared the results obtained after 4 weeks to those obtained after 12 weeks. The EPC/PLGA/CPM group showed significant differences in its BV/TV (31.04 ± 2.83, 44.33 ± 1.2, respectively, *p* = 0.014) and Tb.Th (0.168 ± 0.007, 0.196 ± 0.01, respectively, *p* = 0.048) values over time. These differences were even more significant in the EPC/PLGA group (BV/TV: 27 ± 1.53, 49.02 ± 0.94, respectively, *p* < 0.01; Tb.Th: 0.17 ± 0.005, 0.28 ± 0.012, respectively, *p* < 0.01) (Figure 4b,c).

### 2.5. Histology

We did not notice any inflammatory responses at the transplantation sites in our in vivo experiments. This agrees with previous reports that showed that EPC-transplantation and CPMs do not cause significant inflammatory responses [34,35]. At week 4, the surface of the cartilage in the EPC/PLGA/CPM group was smoother and thicker than that of the other groups. Although there were still some flaws, the newly formed superficial cartilage and subchondral bone could be clearly distinguished under Masson’s trichrome staining. We noticed a fine integration between the regenerative tissue, the preexisting tissue, and the adjacent cartilage and bone. The EPC/PLGA/CPM group also presented glycosaminoglycan (GAG) deposition and oriented collagen alignment. The surface of the cartilage in the EPC/PLGA group was interrupted, and the fibrocartilaginous tissue was mixed with GAG, which is consistent with our previous study [13]. The ED group did not show adequate healing and exhibited fibrous tissues, large residual void spaces, and reduced bone repair.

**Figure 5:**
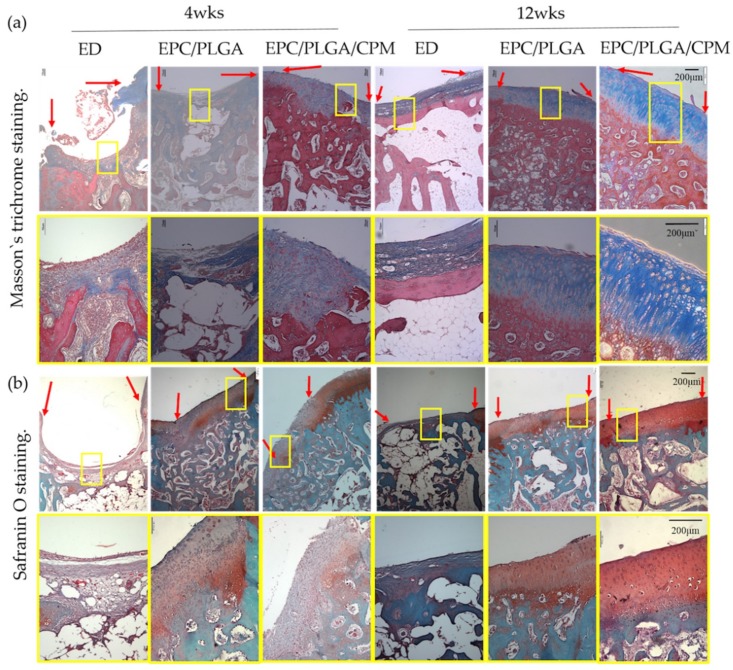
Histology of the different groups. Representative images of histological examinations using (**a**) Masson’s trichrome and (**b**) Safranin O staining. At week 4, the EPC/PLGA/CPM group showed distinctly smooth and thick cartilage and subchondral bone formation. In the EPC/PLGA group, fibrocartilaginous tissue was mixed with a mild cartilaginous matrix. At week 12, the EPC/PLGA/CPM group showed regenerative tissue in the defect sites that was almost exclusively hyaline-like cartilage with a regular surface and was well-integrated with the preexisting tissue. In the EPC/PLGA group, more abundant cartilage formation and bone regeneration than at 4 weeks were apparent, but the osteochondral junction between the regenerated tissue with the host tissue was apparent. In the ED group, we observed fibrous tissue formation and discontinuous subchondral bone reconstruction. A yellow square denotes the magnification scale. A red arrow shows the border of the repaired tissue. Scale bar 200 μm.

After 12 weeks, the PLGA scaffolds were almost degraded, and we observed the formation of new tissue that regenerated the cartilage and bone at the osteochondral defect in the EPC/PLGA/CPM group. The bright red safranin O staining was distinctive and identified the formation of an abundant cartilage matrix. The cell alignments were markedly similar to that of the native cartilage. The regenerative tissue in the defect sites was almost exclusively hyaline-like cartilage with a regular surface and was well-integrated with the preexisting tissue. In the EPC/PLGA group, we noticed a more abundant cartilage formation and bone regeneration than at 4 weeks, but the osteochondral junction between the regenerated and preexisting tissue was apparent. In the ED group, we found fibrous tissue formation instead of cartilage and a discontinuous subchondral bone reconstruction (Figure 5).

**Figure 6:**
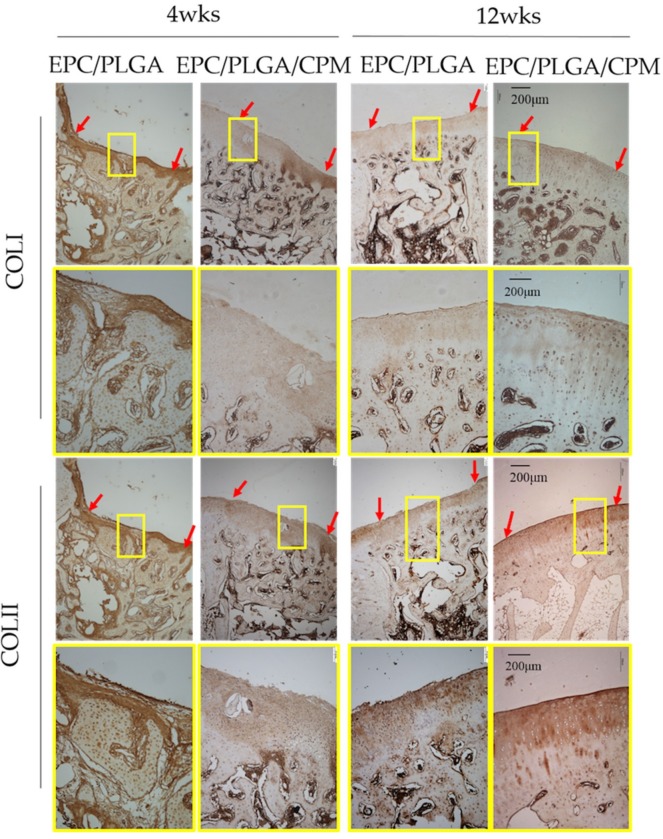
Representative images of specific proteins within the defect sites detected by Immunohistochemistry (IHC) staining. Four weeks after implantation, collagen type II (COLII) was present in the cartilage of the EPC/PLGA/CPM group. In the EPC/PLGA group, the PLGA scaffold was not degraded, and the surface was irregular. Twelve weeks after implantation, the COLII and collagen type I (COLI) staining were more distinctive in the EPC/PLGA/CPM group than in the EPC/PLGA group. A yellow square denotes the magnification scale. A red arrow shows the border of the repaired tissue. Scale bar 200 μm.

**Figure 7:**
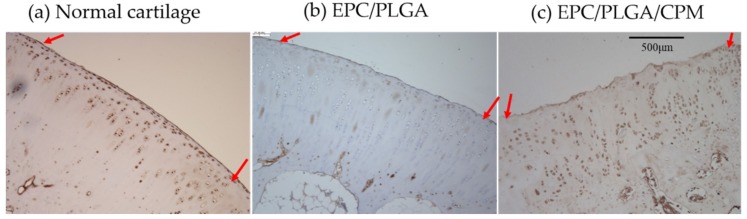
Representative images of superficial proteins in the defect sites detected by IHC staining. Lubricin immunostaining in dissected cartilage samples was visualized throughout the extracellular matrix of all groups. Normal cartilage (**a**): A very strong lubricin staining (intensity of staining (IS) = 4; extent score (ES): ++++) was observed; EPC/PLGA group (**b**): Weak lubricin immunoreactivity (IS = 1; ES: +); EPC/PLGA/CPM group (**c**): Robust lubricin staining (IS = 4; ES: ++++). A red arrow shows the border of the repaired tissue. Scale bar 500 μm.

We performed immunohistochemical analysis of the regenerated tissue to assess collagen type II (COLII), collagen type I (COLI), and lubricin expression. At 4 weeks, the undegraded PLGA scaffold was still apparent. In the EPC/PLGA group, the surface of the cartilage was irregular, and PLGA remained in the subchondral bone. However, in the EPC/PLGA/CPM group, we observed COLII in the cartilage and COLI in the subchondral bone. Although there were still some flaws, the cartilage and subchondral bone regeneration were almost complete. At 12 weeks, the PLGA was completely degraded. In the EPC/PLGA group, we observed a smoother and thicker cartilage surface and distinctive COLII and COLI expression in the cartilage and subchondral bone, respectively, (Figure 6); lubricin expression was weak or absent (intensity of staining (IS) = 1; extent score (ES): +) (Figure 7). The COLII and COLI stainings were more distinctive in the EPC/PLGA/CPM group (Figure 6), and a robust lubricin staining (IS = 4; ES: ++++) was observed in the superficial zone of the articular cartilage (Figure 7).

## 3. Discussion

Currently, the treatments in osteochondral regenerative medicine mainly consist of different combinations of temporary 3D constructs, additional growth factors, external physical stimuli, and exogenous cells. In this study, we used a three-factor combination to enhance cartilage repair: EPCs that could be extracted from peripheral blood, amplified in vitro, and applied to the microenvironment; a PLGA scaffold that supplied mechanical support; and early CPM that provided external physical stimuli to stimulate the therapeutic cells from bone marrow or synovial cells.

Our histological data (Figure 5) showed that 4 weeks after transplantation, the EPC/PLGA/CPM group had succeeded in establishing the architecture of the osteochondral tissue and had a smooth surface, it also presented a moderate amount of GAG. Furthermore, our results showed that CPM induced lubricin expression by the superficial cartilage 12 weeks after the EPC/PLGA construct transplantation. The groups that did not undergo CPM expressed no lubricin. Moreover, the IVIS data (Figure 2) showed that the bioluminescence remained close to the defect site in the CPM group. This might be because CPM induced the cells to proliferate in the defect sites, explaining why the early osteochondral regeneration at 4 weeks and lubricin expression at 12 weeks were only observed in the EPC/PLGA/CPM group.

The EPC/PLGA/CPM group had a similar BV/TV ratio and Tb.Th to those of the sham group, while the group without CPM intervention exhibited significantly higher values than those of the sham group, which is consistent with our previous study [13]. These data suggest that early CPM prevents joint stiffness, edema formation, and subchondral bone overgrowth after surgery. Furthermore, CPM triggers the surrounding therapeutic stem cells to respond to the microenvironment created by the EPCs, thus promoting chondrogenesis [13], osteogenesis [21,36], and angiogenesis [37,38] and remedying osteochondral defects at early stages.

Various strategies have been adopted to regenerate the complex osteochondral tissue. For example, multi-layered scaffolds [39] could support the host reparative response, but have the risk of delamination [40,41]. Cell-based therapy is an advanced orthobiological technology for cartilage regeneration, but its potential remains controversial. According to a previous study, EPC/PLGA constructs offer a vessel-rich microenvironment for nutrition and support in the tissue of the regenerating joint and provide the paracrine growth factors, transforming growth factor beta 2 (TGF-β2) and transforming growth factor beta 3 (TGF-β3), that mediate the responses of cartilage and bone regeneration [13]. Previous results demonstrated that transplantation with highly angiogenic EPCs induces hyaline cartilage and subchondral regeneration in osteochondral defects. However, EPCs that originate in the bone marrow still lead to a high BV/TV ratio [13] and bone formation during cartilage repair. In the present study, we observed that CPM induced the expression of signals that maintained chondrogenesis during osteochondral regeneration.

Early CPM rehabilitation was investigated over active treadmill exercise due to its potential to decrease the risk of posttraumatic osteoarthritis and to avoid cruciate ligament reconstruction [42]. Also, the evidence indicated that proper rehabilitation exercises benefit the knee joint restoration and improve collagen formation, GAG expression, and bone remodeling [43,44,45]. A previous report studied a similar position to that of our study and also demonstrated that the femoral medial condyle endured continuous sliding against the meniscus and tibial cartilage during CPM and that the synthesis of *PRG4* in chondrocytes was higher with CPM than without [26].

The cartilage from the EPC/PLGA/CPM joints expressed lubricin despite the cell misalignment shown by the IHC staining, probably because the transplanted cell-construct could not completely regenerate to match the original tissue. However, the degree of lubricin expression in this study was similar to that of a previous study [33]. CPM promoted the maintenance of the microenvironment created by the EPCs and induced chondrogenesis via migration of the host cartilage stem/progenitor cells or the bone marrow mesenchymal stem cells to the subchondral bone. However, the dynamic stimuli would induce lubricin expression at depths of 200–400 μm from the articular surface [46]. Ogawa et al. demonstrated that mechanical motion induced lubricin in the articular cartilage via the prostaglandin E_2_ (PGE2), parathyroid hormone-related peptide (PTHrP), and adenosine triphosphate (ATP) signaling pathways [47]. We suggested that CPM had the potential to induce lubricin expression in superficial cartilage in vivo. The Masson’s trichrome staining (Figure 5) showed that the EPC/PLGA/CPM group secreted some components of the extracellular matrix and enhanced the formation of new subchondral bone after 4 weeks. At that time, the EPC/PLGA group still exhibited non-regenerated regions in the defect. This suggests that the physical stimuli facilitate the influx of host cells into the implanted construct during scaffold degradation [34].

In this study, we used the salt-leaching method to fabricate the three-dimensional PLGA scaffold. This procedure has advantages such as the feasibility to control pore size, the mechanical rigidity, and the degradation rate according to the polylactic acid (PLA)-to-polyglycolic acid (PGA)-ratio. Following this procedure, EPCs could lie firmly in the pores of the PLGA structure during CPM. Previous studies have demonstrated that the degradation half-time of PLGA scaffolds in vitro was around 3–4 weeks [48] and that the scaffolds should be totally degraded after 12 weeks. Our histology data confirmed that the PLGA scaffold degraded gradually while newly formed subchondral bone and cartilage-like tissue accumulated at the wound, suggesting that the structure attracted progenitor cells in the blood and bone marrow. The architecture of the PLGA scaffold was fixed to the defect without using periosteum as a cover, and the cells were easily inoculated without leakage. The structure also provided a topographical cue to promote cell migration, attachment, and proliferation and tissue regeneration.

This study has some limitations. First, we did not perform a biomechanical test to examine the mechanical properties of the regenerated cartilage and the friction coefficient on its surface. To minimize the number of sacrificed animals, we focused on the regeneration degree and the biochemical and molecular analyses of the regenerated tissue. Second, we did not test the EPC/PLGA construct with a CPM bioreactor to investigate the degree of chondrogenesis and osteogenesis ex-vivo and contrast them to the data in vivo. However, the process performed ex-vivo could not mimic the in vivo environment due to the lack of growth factors, so we only examined the regenerative effects directly on our rabbit model.

To our knowledge, this is the first study demonstrating that transplantation of an EPC/PLGA construct combined with early CPM can regenerate the cartilage in an appropriate superficial zone. CPM is used after orthopedic surgical procedures for patient rehabilitation. Even starting CPM the day after surgery, the EPC/PLGA construct remained firmly at the defect sites for 4 weeks (Figure 2). CPM did not disturb the microenvironment created by the EPC/PLGA construct. It is likely that the growth of the transplanted EPCs also avoided PLGA leakage during CPM (Figure 2).

Chondrogenesis maintenance is a determinant to cartilage regeneration. The most promising cell source, mesenchymal stem cells (MSCs), are prone to forming hypertrophic cartilage and undergoing endochondral bone formation [49]. Various strategies have been adopted to avoid this situation such as hypoxia [50] and co-culture [51,52]. In this study, the mechanical stimuli provided by CPM not only prevented endochondral bone formation but also maintained chondrogenesis during osteochondral regeneration after the transplantation of the EPC/PLGA construct.

## 4. Materials and Methods

### 4.1. Isolation and Culture of EPCs

Blood from New Zealand white rabbits was obtained via the peripheral ear artery (10 mL/kg), as described previously [13]. We used the method of density gradient centrifugation to isolate monocytes. Isolated monocytes were washed and incubated with endothelial growth medium (EGM)-2 (Lonza, Basel, Switzerland). The medium was changed twice a week. After 7 days of culture, the adherent cells (known as early EPCs) were harvested for transplantation.

### 4.2. Tracking the Implanted EPCs

To track the implanted EPCs in vivo, they were stained with chloromethyl-benzamidodialkylcarbocyanine (CM-DiI; Molecular Probes, Carlsbad, CA, USA) [53] before transplantation. The red fluorescence from CM-Dil was visible in the defect zones, and the eluent was monitored by fluorescence at excitation at 553 nm/emission 570 nm using Xenogen IVIS^®^ Spectrum Noninvasive Quantitative Molecular Imaging System (PerkinElmer Inc., Waltham, MA) 4 and 12 weeks after implantation.

### 4.3. Fabrication of Porous PLGA and EPC/PLGA Scaffolds

All procedures were performed as previously described [13]. PLGA (lactide:glycolide ratio 85:15 (85/15), molecular weight: 50,000–75,000) was used to fabricate the scaffolds using the salt-leaching technique. Briefly, sodium chloride mixed with 20% *weight*/*volume* PLGA chloroform solution was poured into cylindrical molds and lyophilized for 1 day. The sponges were immersed in deionized water to dissolve the porogen and lyophilized again to give them shape (final dimensions, 3 mm in height and 3 mm in diameter). EPCs were resuspended in medium at 5.0 × 10^5^ cells/mL and seeded into PLGA scaffolds with syringes. Fresh medium was supplied after 2 h, and the scaffold was incubated for 1 day before transplantation.

### 4.4. Animal Procedures

This animal study was approved 9 January 2014 by the Institutional Animal Care and Use Committee of National Cheng Kung University (No. 103208). Animal care and all experiments were conducted following the guidelines. A rabbit osteochondral defect model was created on the weight-bearing zone of the medial femoral condyle as previously described [34]. Twenty (40 knees in total) 5-month-old New Zealand white male rabbits (2–3 kg) were purchased from Livestock Research Institute, Taiwan. The animals were anesthetized by subcutaneous injection of Zoletil 50 (10–15 mg/kg) and Rompun 20 (10–15 mg/kg). The rabbits were randomly divided into one of four treatment groups: empty defect (ED) (N = 12), sham (N = 4), EPC/PLGA scaffold (N = 12), and EPC/PLGA/CPM (N = 12). In the sham group, we only performed a surgical incision in both knee joints. In all other groups, we performed the incision and created an osteochondral defect. In the EPC/PLGA and EPC/PLGA/CPM groups, the structures were gently inserted by pressed fitting. After the surgical procedure, the patella was relocated, and the femur was stabilized by flexion and extension movements to ensure scaffold fixation. The animals were housed under standardized cage conditions, a 12 h light-dark cycle, and allowed food and water *ad libitum*. An antibiotic (5 mg/kg, Enrofloxacin, Bayer, Leverkusen, Germany) and analgesic (2–10 mg/kg, Meperidine, FDA controlled drug pharmaceutical factory, New Taipei city, Taiwan) were applied for 3 days. We put neck collars on the rabbits to prevent biting. The rabbits were euthanized after 4 or 12 weeks with an overdose of anesthesia and an intravenous injection of potassium chloride. The repaired osteochondral tissues were extracted for further examination.

**Figure 8:**
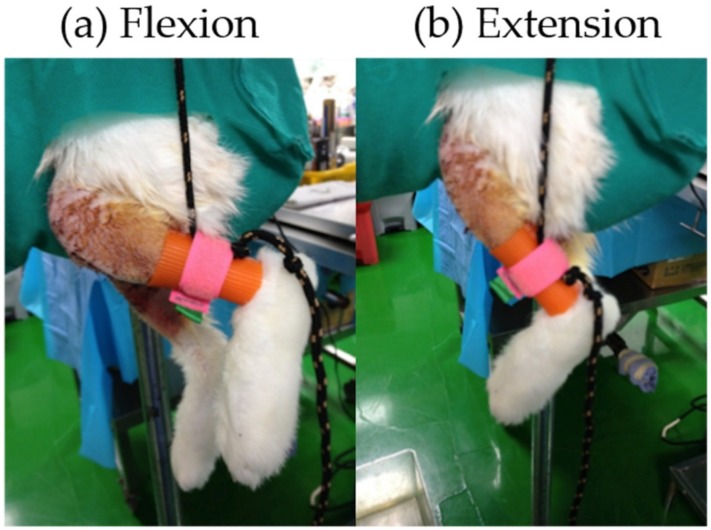
Short-term CPM treatment. (**a**) Flexion (**b**) Extension.

After the surgery, we applied CPM on the operated knee joints of the EPC/PLGA/CPM group as previously described [34]. Briefly, the day after the surgery, a passive motion was applied to the knee of a rabbit without anesthesia to monitor stress and pain tolerance. The CPM machine induced flexion/extension (60–130 degrees) (Figure 8) for 15 min/day; for a week after CPM management, rabbits were allowed free cage activity. The ED, sham, and EPC/PLGA groups were allowed free activity in the cage.

### 4.5. Macroscopic Assessment

The rabbits were euthanized after 4 and 12 weeks (ED, EPC/PLGA, and EPC/PLGA/CPM: 6 knees each; Sham: 4 knees at each time point) for macroscopic examinations. The appearance of regenerated tissue in each operative site was blindly assessed by two individuals using the modified Wayne’s grading scale scoring system (Supplementary Table S1) [34].

### 4.6. Micro-CT Evaluations

The dissected femoral condyles were analyzed using a high-resolution micro-computed tomography (micro-CT) 1076 scanner (Skyscan, Kontich, Belgium) to qualitatively and quantitatively asses the bone regeneration in the tissues of the different groups. The scanning parameters were set according to our previous study [34]. The images were reconstructed and analyzed using the Skyscan software package (https://www.bruker.com/service/support-upgrades/software-downloads/micro-ct/library.html). The volume and diameter of the bone growth were assessed by measuring the bone volume per tissue volume (BV/TV) and trabecular thickness (Tb.Th).

### 4.7. Staining, Histology Scores, and Immunostaining

After the micro-CT scanning procedure, the dissected femoral condyle was fixed with 10% neutral buffered formalin and then decalcified in 10% formic acid/ phosphate-buffered saline (PBS). The decalcified samples were embedded in paraffin wax, and 4-μm microsections in the coronary plane were prepared. The histological slides were stained with Safranin O–fast green and Masson’s trichrome to assess the glycosaminoglycan (GAG) and collagen alignment, respectively. Immunohistochemical staining for COLI (fibrocartilage), COLII (hyaline cartilage), and lubricin (superficial zone protein) were performed using the rabbit/mouse horseradish peroxidase (HRP)/3,3′-Diaminobenzidine (DAB) polymer detection (BioSB, Santa Barbara, CA, USA) kit.

### 4.8. Evaluation of Immunohistochemistry

The lubricin staining was determined as negative or positive. Brown chromogen detection on the edge of the hematoxylin-stained cell nucleus was defined as positive. The images of staining intensity and the proportion of immunopositive cells from the sections were blindly evaluated and scored by three investigators. The intensity of staining (IS) was ranked from 0–4, as previously described [33,54]: no detectable staining (0), weak staining (1), moderate staining (2), strong staining (3), very strong staining (4). The percentage of lubricin immunopositive cells (Extent Score = ES) was divided into five categories: <5% (0); 5–30% (+); 31–50% (++); 51–75% (+++), and >75% (++++). Normal articular cartilage was used as a positive control.

### 4.9. Statistical Analysis

All data are shown as the mean ± standard error of the mean (SEM). Statistical analyses were performed using the SPSS v. 17.0 software package (IBM, Armonk, NY, USA). For comparisons between groups, we used the Mann–Whitney U-test. For comparisons between different time-points, we used generalized estimating equations (GEE) [55]. *p* < 0.05 was considered statistically significance.

## 5. Conclusions

In conclusion, we demonstrated that early CPM could act in combination with the EPC/PLGA construct and maintain the in situ microenvironment for the regeneration of osteochondral regions providing tissue-specific regeneration and an appropriate integration between the osteochondral and the chondroprotective surfaces of the cartilage. The procedure presented in this study regenerated the cartilage, including the superficial zone, in a rabbit osteochondral defect model and further prevented cartilage degeneration without the addition of exogenous growth factors. Rehabilitation post-operatory therapies such as CPM are beneficial and should be implemented as routine procedures to promote and maintain chondrogenesis during the clinical treatment of articular osteochondral defects.

## Supplementary Materials

Supplementary materials can be found at http://www.mdpi.com/1422-0067/20/2/259/s1. Table S1. Modified Wayne’s grading scale scoring system for gross appearance.

## Abbreviations

ACI	Autologous chondrocyte implantation
COLI	Collagen type I
COLII	Collagen type II
CPM	Continuous passive motion
ED	Empty defect
EGM	Endothelial growth medium
EPC	Endothelial progenitor cell
ES	Extent score
GAG	Glycosaminoglycan
GEE	Generalized estimating equations
IHC	Immunohistochemistry
IS	Intensity of staining
MACI	Matrix-induced ACI
MSC	Mesenchymal stem cell
OA	Osteoarthritis
OCT	Osteochondral transplantation
ROI	Region of interest
SEM	Standard error of the mean
TKA	Total knee arthroplasty
