# Supplementary material for: Continuous Passive Motion Promotes and Maintains Chondrogenesis in Autologous Endothelial Progenitor Cell-Loaded Porous PLGA Scaffolds during Osteochondral Defect Repair in a Rabbit Model
Source: Int J Mol Sci. 2019 Jan 10;20(2):259. doi: 10.3390/ijms20020259 (PMC6358980; doi:10.3390/ijms20020259)
Supplement: Supplementary file 1 [file ijms-20-00259-s001.pdf]

Supplementary Table 1. Modified Wayne's grading scale scoring system for gross appearance.

Macroscopic Appearance	Description	Points
1. Coverage	>75% filled	4
	50–75% filled	3
	25–50% filled	2
	<25% filled	1
	0% filled	0
2. Tissue Color	Normal/whitish	4
	25% yellow/brown/reddish/white	3
	50% yellow/brown/reddish/white	2
	75% yellow/brown/reddish/white	1
	100% yellow/brown/reddish/white	0
3. Surface (smoothness level)	Normal	4
	Smooth but raised	3
	25–50% irregular	2
	50–75% irregular	1
	>75% irregular	0
Total score	For full evaluations	12
